# Supplementary material for: Therapeutic Patterns and Clinical Outcomes in Limited Disease Small Cell Lung Cancer: A Decade of Analysis at a Tertiary Cancer Center
Source: Cancers (Basel). 2024 May 21;16(11):1953. doi: 10.3390/cancers16111953 (PMC11171404; doi:10.3390/cancers16111953)
Supplement: Supplementary file 1 [file cancers-16-01953-s001.zip › cancers-2970769-supplementary.pdf]

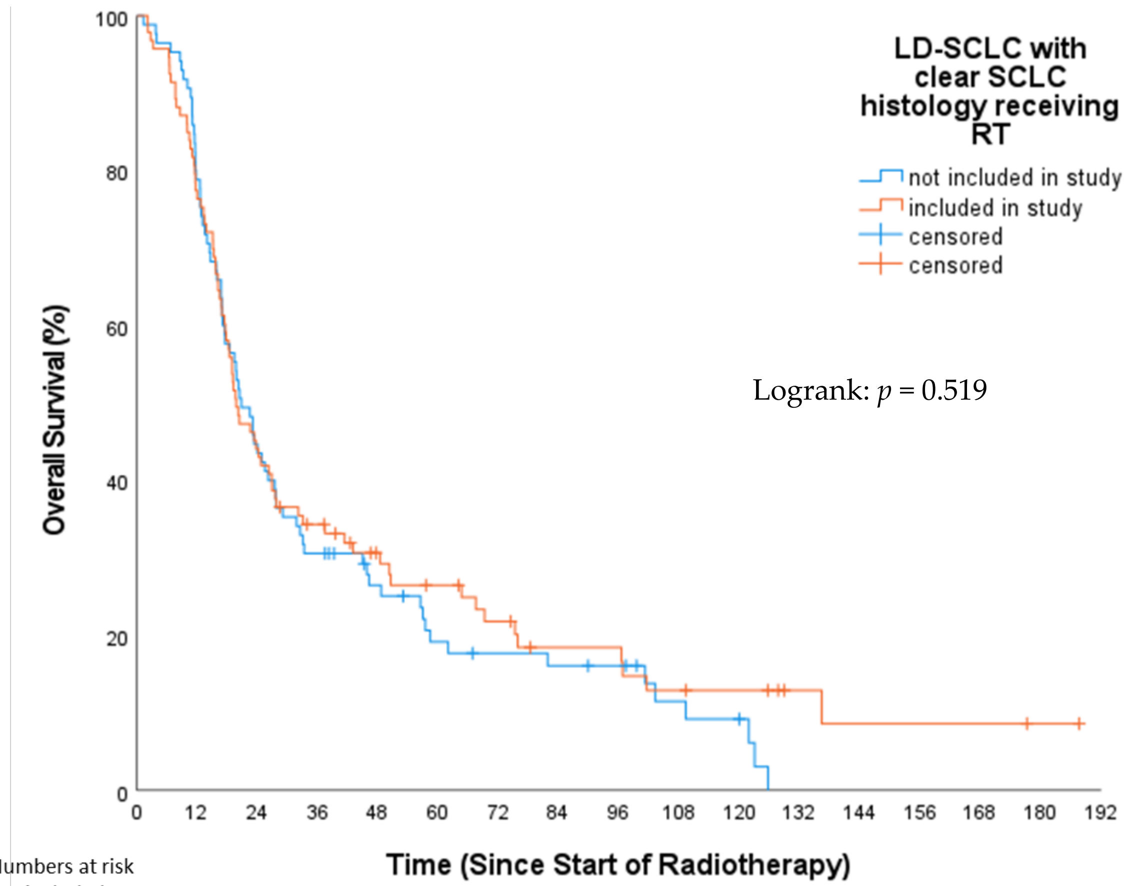

Numbers at risk																
not included:	85	67	38	26	19	13	11	10	9	5	3	0	0	0	0	0
included:	93	72	41	30	22	18	14	10	10	7	6	3	2	2	2	1

Figure S1. OS Comparison: Ineligible vs. Included SCLC Patients Undergoing Radiotherapy. This figure illustrates the overall survival (OS) data of patients with clear SCLC histology who underwent radiotherapy. It is divided into 85 ineligible patients (no clear chemotherapy treatment or no chemotherapy (n = 75); thoracic resection before radiotherapy (n = 5); only PCI (n = 5)) and 93 patients who were finally included in the study.
